# Direct RT-PCR amplification of SARS-CoV-2 from clinical samples using a concentrated viral lysis-amplification buffer prepared with IGEPAL-630

**DOI:** 10.1038/s41598-021-93333-2

**Published:** 2021-07-09

**Authors:** Alejandro Castellanos-Gonzalez, Thomas R. Shelite, Nicole Lloyd, Aygul Sadiqova, Ren Ping, Natalie Williams-Bouyer, Peter C. Melby, Bruno L. Travi

**Affiliations:** 1 Infectious Diseases Division, Internal Medicine Department, University of Texas Medical Branch, Galveston, TX USA; 2 Infectious Diseases Division, Pathology Department, University of Texas Medical Branch, Galveston, TX USA

**Keywords:** Infectious diseases, Viral infection

## Abstract

The pandemic of 2019 caused by the novel coronavirus (SARS-CoV-2) is still rapidly spreading worldwide. Nucleic acid amplification serves as the gold standard method for confirmation of COVID-19 infection. However, challenges faced for diagnostic laboratories from undeveloped countries includes shortage of kits and supplies to purify viral RNA. Therefore, it is urgent to validate alternative nucleic acid isolation methods for SARS-CoV-2. Our results demonstrate that a concentrated viral lysis amplification buffer (vLAB) prepared with the nonionic detergent IGEPAL enables qualitative detection of SARS-CoV-2 by direct Reverse Transcriptase-Polymerase Chain Reaction (dRT-PCR). Furthermore, vLAB was effective in inactivating SARS-CoV-2. Since this method is inexpensive and no RNA purification equipment or additional cDNA synthesis is required, this dRT-PCR with vLAB should be considered as an alternative method for qualitative detection of SARS-CoV-2.

## Introduction

Severe acute respiratory syndrome Coronavirus-2 (SARS-CoV-2) has generated a global pandemic due its rapid spread and fatal progression of the coronavirus infection (COVID-19)^1^. Early diagnosis of COVID-19 is crucial for disease treatment and control^2^. Many symptoms of SARS-CoV-2 overlap with other respiratory illnesses, so confirmation of the presence of the virus is necessary for accurate diagnosis^3^. Currently, the reverse transcriptase amplification (RT-PCR) method is the gold standard for the diagnosis of SARS-CoV-2 in respiratory samples^4^. In early 2020, the Centers for Disease Control and Prevention (CDC) in the USA developed an RT-PCR assay for detection of SARS-CoV-2^5^, and a few weeks later the Federal Drug Administration (FDA) authorized this assay for emergency use^5,6^. The RT-PCR primers described in the CDC protocol showed high sensitivity (600–1200 viral genome copies/mL) and specificity^7^ so are routinely used in many laboratories around the world. This assay requires RNA purification and several purification kits have been validated. However, the high demand for RNA purification kits has resulted in worldwide shortages that have affected several diagnostic laboratories, especially in undeveloped countries. Therefore, it is necessary to validate new RNA isolation methods useful for COVID detection.

Direct PCR (dPCR) is a strategy to conduct DNA/RNA amplification directly from a sample without performing DNA/RNA isolation and purification steps. As such, this technique greatly reduces sample processing time^8^. Detergents included in dPCR buffers induce cellular lysis with release of nucleic acids, which allows robust amplification despite the presence of PCR inhibitors often found in crude samples^9–12^. IGEPAL CA-630 is a nonionic, non-denaturing detergent that has been successfully used for dRT-PCR amplification^10,13^. However, IGEPAL has not been evaluated for inactivation of SARS-CoV-2 and neither tested for dRT-PCR detection following CDC protocol. Here, we used IGEPAL to prepare a viral lysis-amplification buffer (vLAB) and demonstrated that this buffer is suitable for the qualitative detection of SARS-CoV-2 by dRT-PCR in clinical samples following the CDC protocol.

## Methods

All methods were carried out in accordance with guidelines and regulations in methods section. Experimental protocols including the use of clinical samples were approved by the Institutional Review Board (IRB) at University of Texas Medical Branch (UTMB). For some experiments we used samples (otherwise discarded) diagnosed in UTMB clinical laboratory. The de-identified samples were obtained from patients during standard of care procedures in which the investigator had no interaction in obtaining samples (Waiver of Informed Consent [45 CFR 46.116] for collected samples was approved by IRB at UTMB). Inactivation protocol, viability testing protocol, and in-house generated data was approved by the subcommittee of the Institutional Biosafety Committee (No. 14010) at UTMB.

### Viral lysis-amplification buffer (vLAB)

For dRT-PCR experiments, we tested clinical samples and RNA spiked in water. All clinical samples were treated as described below with a concentrated solution containing IGEPAL (MilliporeSigma, St. Louis, MO) called viral lysis-amplification buffer (vLAB). The concentrated 10X buffer was prepared as follows: (25 mL vLAB) 100 mM Tris–HCl pH 7.4, IGEPAL CA-630 0.5%, NaCl 150 mM. One mL of this solution was supplemented with 2.5 mL of BSA 100X (New England Biolabs, Ipswich, MA). vLAB was filtered through a 0.22 µm membrane and then aliquoted and stored at room temperature until use.

### SARS-CoV-2 RNA and clinical samples

All viral RNA used in this work was obtained from the World Reference Center for Emerging Viruses and Arboviruses (WRCEVA) at University of Texas Medical Branch, Galveston Texas. For initial experiments, we tested dRT-PCR performance with RNA spiked in water or vLAB, and then we prepared tenfold dilutions (ranging from 1 × 10^7^ to 1 × 10^0^) to conduct dRT-PCR amplification as described below. For some experiments, we tested dRT-PCR performance with spiked RNA samples incubated under different temperatures: 70 °C for 10 min and 95 °C for 2 min. To evaluate vLAB performance in clinical samples, we used de-identified discarded nasopharyngeal swabs that had been collected for COVID-19 diagnostic testing in the UTMB clinical laboratory. The nasopharyngeal swabs were placed in 3 mL of Viral Transport Medium (VTM; Hanks Balanced Salt Solution, 2% FBS, 100 µg/mL gentamicin, and 0.5 µg/mL Amphotericin B), following standard protocols for COVID-19 sample collection recommended by CDC. Clinical samples were heat inactivated and then transported to our BSL-2 enhanced laboratory and tested immediately or stored at − 80 °C until use. Negative controls: negative clinical samples and unrelated pathogens in VTM were obtained from UTMB clinical laboratory. Unrelated viral RNA (MERS and SARS) was obtained from CDC-SARS-CoV-2 detection kit (IDT, Coralville Iowa). All RT-PCR experiments with viral RNA and clinical samples were conducted in triplicate and results are shown as averages of CT values.

### Inactivation of SARS-Cov2 in vLAB

We infected monolayers of Vero E6 cells with mNG-SARS-Cov2 diluted in vLAB to evaluate virus inactivation. Before the infection, 10 μL of mNG-SARS-CoV-2 (1 × 10^7^ PFU/mL) were diluted with 90 μL of vLAB. Some samples were incubated at room temperature (RT) for 10 min, and other samples were incubated at RT for 20 min. Post-incubation, 10 μL from each sample or MEM media (negative control) were added to each well of a Falcon 12-well plate seeded with 5 × 10^4^ cells/well in 2 mL Gibco 1X MEM with 10% fetal bovine serum and gentamycin. Each condition (negative control, positive control, 10 min, and 20 min) was run in triplicate. Plates were incubated at 37 °C with 5% CO_2_ for 3 days and then analyzed by fluorescent microscopy. To confirm inactivation, from the first passage plate, 10 μL of supernatant was inoculated in triplicate onto another 12-well plate seeded with 5 × 10^4^ cells/well in 2 mL of 10% 1X MEM. This plate was incubated again at 37 °C for 3 days and then analyzed by microscopy as before. Inactivation experiments were conducted in BSL-3 facilities (Dr. Bukreyev’s laboratory at UTMB) and the inactivation protocol was approved by the Institutional Biosafety Committee at UTMB dPCR from pure SARS-Cov-2 RNA and inactivated cells.

For these experiments, we used SARS-Cov-2 RNA (ranging from 0–50,000 copies) obtained from WRCEVA or supernatants of infected cells previously inactivated with vLAB as described above. We conducted all RT-PCR amplifications with the Quantabio RT-qPCR Tough Mix Kit and we used primers and probes for nucleocapsid (N1 and N2) designed by CDC included in the 2019-nCov CDC EUA Kit, 1000 rxn (Integrated DNA Technologies, Coralville, Iowa). After adding template (2 µL) to RT-PCR master mix, reaction (20 µL total volume) was transferred to PCR 96 well plates (Applied Biosystems, Foster City CA) and amplification was conducted in a 7500 Fast Real-Time PCR System (Applied Biosystem) using the following conditions: 50 °C for 15 min, 95 °C for 5 min, then 45 cycles of 95° C 3 s and 55 °C for 45 s. All clinical samples and controls were tested in triplicate.

### dRT-PCR and RT-PCR from clinical samples

We evaluated dRT-PCR amplification in vLAB and for some experiments we compared dRT-PCR vs standard RT-PCR for SARS-CoV-2. For direct amplification, we used RT-PCR conditions, reagents and clinical samples diluted as described before. For standard amplification we purified SARS-CoV-2 RNA using QIAamp Viral RNA Mini Kit (Qiagen, Valencia CA) following vendor protocol provided by WRCEVA. For clinical samples, we used 100µL in VTM treated with Trizol followed by modified chloroform separation and RNA isolation using the Qiagen RNeasy Mini Kit (Qiagen). The cDNA synthesis was carried out using the iScript Select cDNA Synthesis Kit (Bio-Rad) following the manufacturer’s protocol or the purified RNA was stored at − 80 °C until use. All clinical samples were previously analyzed in the clinical diagnostic laboratory at UTMB. For SARS-CoV-2 detection, the laboratory used the Panther Fusion System. The Fusion SARS-CoV-2 assay involves the following steps: sample lysis, nucleic acid capture, elution transfer, and multiplex RT-PCR. Nucleic acid capture and elution takes place in a single tube on the Panther Fusion system. The eluate is transferred to the Panther Fusion system reaction tube containing the assay reagents. Multiplex RT-PCR is then performed for the eluted nucleic acid on the Panther Fusion system. The Panther Fusion SARS-CoV-2 assay amplifies and detects two conserved regions of the ORF1ab gene in the same fluorescence channel, ORF1ab Region 1 ORF1ab Region 2. For dRT-PCR and RT-PCR experiments conducted in our laboratory, we used PCR conditions described before and 2 µl of VTM diluted in vLAB 10X or eluted RNA (purified with QIAGEN columns).

## Results

### SARS-CoV-2 RNA amplification in v-LAB

**Figure 1:**
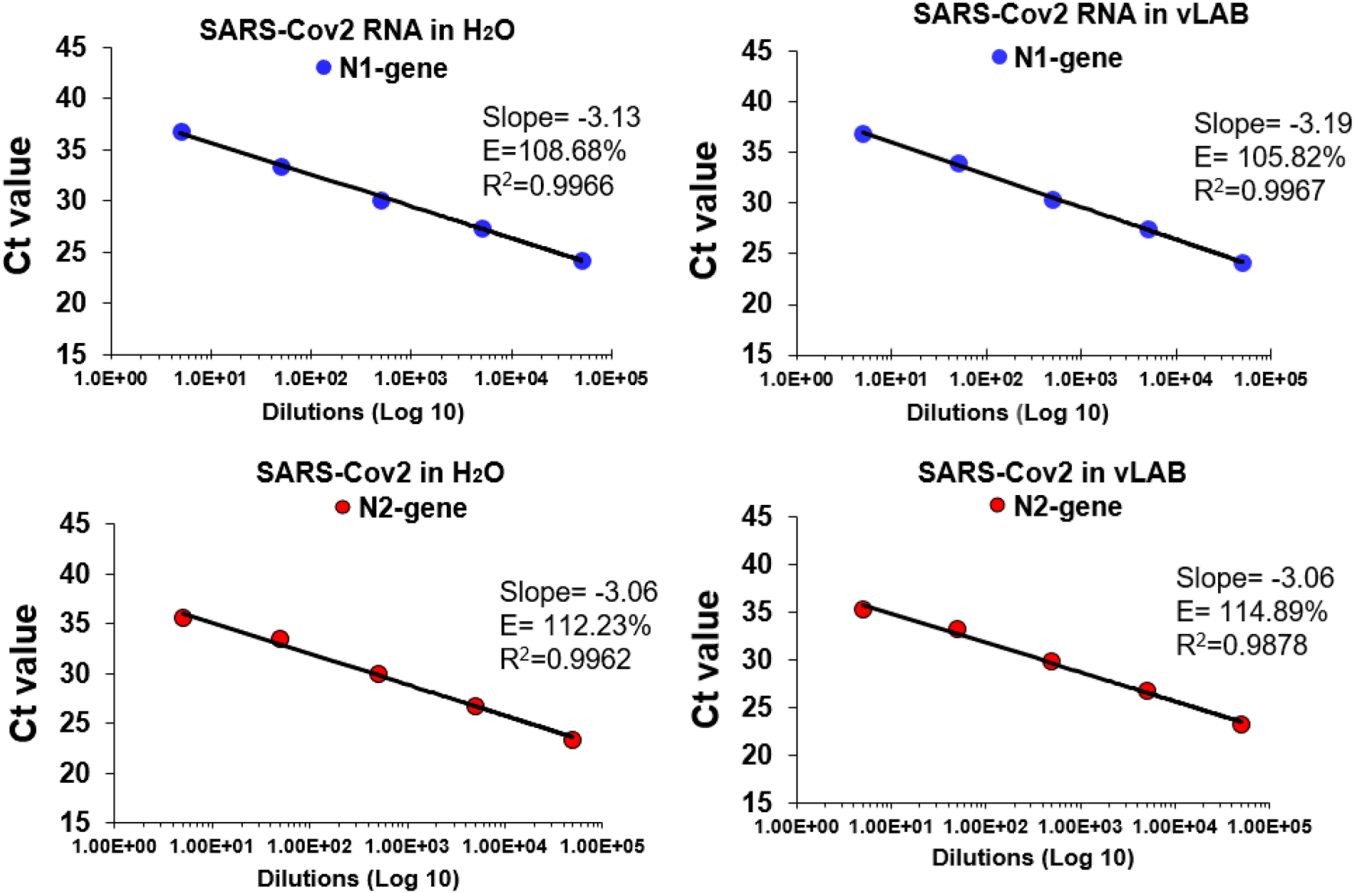
PCR efficiency of SARS-CoV-2 RNA in vLAB. PCR efficiency (E) for N1 and N2 gene was assessed using a duplicate tenfold dilution series of SARS-CoV-2 viral RNA. Linear regression was performed to obtain the slope and R2. The percentage efficiency was calculated from the slope using the formula E = 100 × (− 1 + 10 − 1/slope). *vLAB* virus Lysis Amplification Buffer.

IGEPAL-630 has been previously used for direct RT-PCR amplification and RNA sequencing^9,10^. Our goal here was to use this reagent to conduct direct amplification in suspected COVID-19 samples. However, is not known if IGEPAL-630 affects SARS-CoV-2 RNA integrity or if this detergent inhibits activity of enzymes included in the RT-PCR COVID detection kits approved by CDC. To adress this question, initially we tested amplification of viral RNA spiked in a viral Lysis Amplification Buffer (vLAB) [0.25% IGEPAL, 150 mM NaCL, Tris 10 mM, BSA 1X] using FDA aproved primers/probes for detection of nucleocapside genes N1 and N2. For these experiments, we used serial dilutions (tenfold) of pure RNA diluted in vLAB or water following CDC protocol. We evaluated RT-PCR performance comparing standard curves of RNA in water vs vLAB. Coefficients of correlation (R^2^) obtained from standard curves showed identical values for N1 and N2 genes in both samples (Fig. 1) and CT values with minimal variation (Supplementary Fig. 1) showing the same limit of detection of 5 copies per reaction, and slope and E value (E = PCR efficiency) also did not show significant differences between samples diluted in vLAB and water. These results demonstrated that vLAB buffer does not affect SARS-Cov2 RNA integrity and does not inhibit PCR reagents during amplification.

### SARS-CoV-2 inactivation in v-LAB

Clinical samples collected in COVID-19 patients are commonly manipulated in BSL-2 laboratories for diagnostic purposes. Therefore, to avoid the risk of exposure of laboratory workers SARS-CoV-2 must first be inactivated^14^. Other groups have demonstrated that detergents like sodium-dodecyl-sulfate (SDS) and Triton-X100 added to guanidinium thiocyanate-lysis buffers can reduce virus infectivity. However, inactivation of SARS-CoV-2 with IGEPAL-630 has not previously been determined.

Thus, here we investigated the effect of vLAB on virus replication using a fluorescent SARS-CoV-2 strain (stable mNeonGreen) to infect Vero E6 cells. For these experiments, virus was diluted in MEM culture medium (positive control) or vLAB and then samples were incubated 10 and 20 min. After incubation, the samples were used to infect cultured cells (Pass 1).

**Figure 2:**
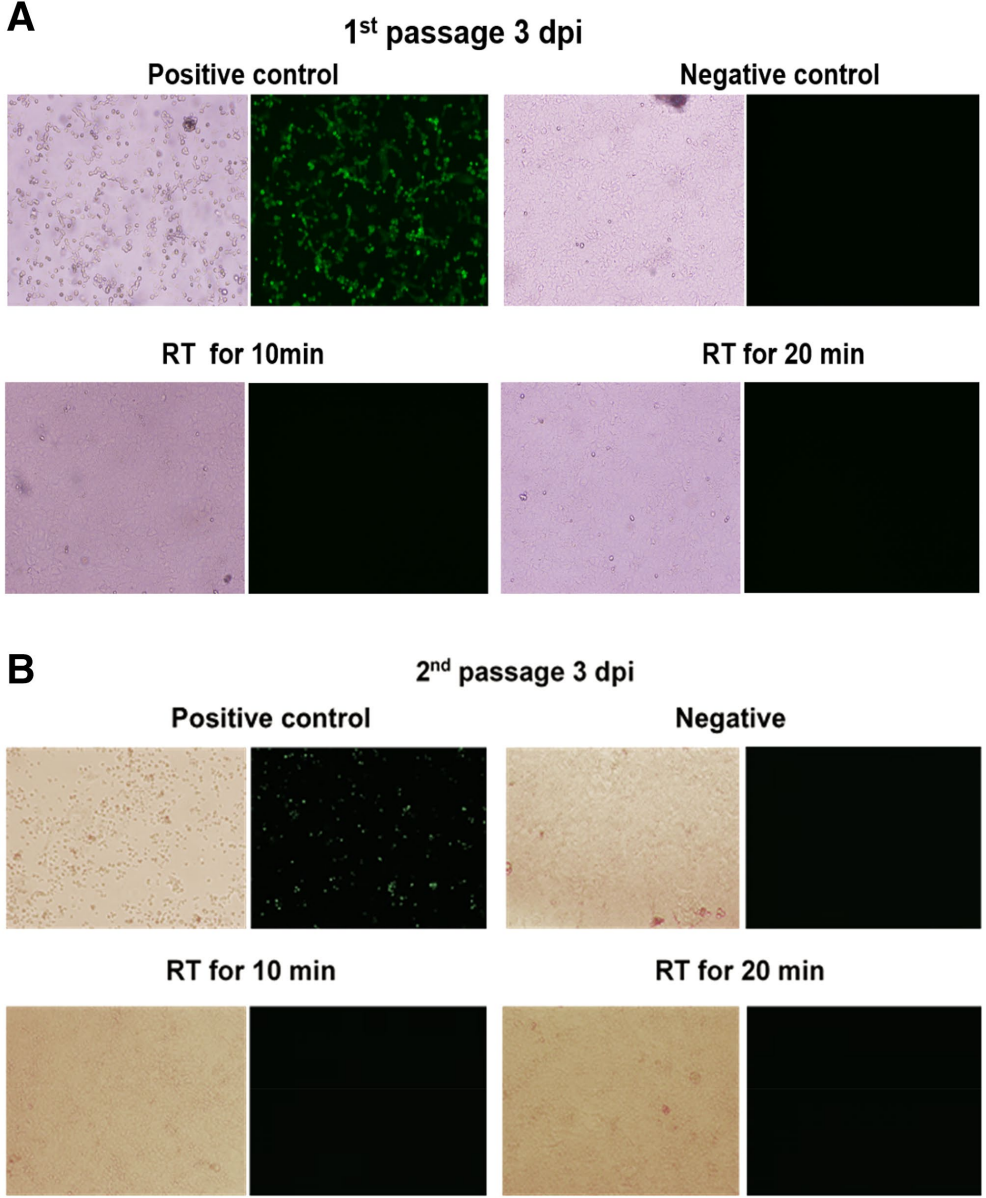
Immunofluorescence images of Vero E6 cells infected with mNG-SARS-CoV-2 diluted in vLAB. Immunofluorescence images of Vero E6 cells infected with stable mNeonGreen (mNG)-SARS-CoV-2 diluted or not in vLAB. Vero E6 Cells monolayers were analyzed 3 days post inoculation (3 dpi). Green cytoplasmic fluorescence is indicative of viral replication. (**A**) Pass1, stable mNeonGreen (mNG)-SARS-CoV-2 virus was diluted in vLAB and then incubated 10 and 20 min at room temperature (RT). Positive control, cells infected with mNG SARS-CoV-2 were diluted only with cell culture media; Negative control = only cell culture media. (**B**) Passage 2nd, supernatants from P1 (10 µL) were used to infect Vero E6 cells.

We analyzed infection on monolayers of Vero E6 cells by fluorescent microscopy after 3 days of inoculation. Microscopy analysis showed no infection in groups of cells exposed to virus incubated with vLAB for 10 and 20 min; only fluorescence in the positive control was observed (Fig. 2A). To confirm viral inactivation, supernatants obtained from pass 1 were used to re-infect Vero E6 cells (Pass 2). However, only untreated samples (positive control) showed viral replication and no infection was detected in samples treated with vLAB (Fig. 2B). Overall, cell culture assays demonstrate that vLAB inactivates SARS-CoV-2, therefore, it should be feasible to conduct molecular diagnostics in BSL-2 labs using clinical samples inactivated with vLAB.

### Direct amplification of SARS-CoV-2

Our initial results demonstrated feasibility to amplify pure RNA diluted in vLAB. However, clinical samples can contain inhibitory molecules that can affect PCR. Therefore, we tested if SARS-CoV-2 from cell lysates can be amplified directly by standard RT-PCR (CDC protocol). For these experiments, we diluted 10 µL of Vero E6 cells infected with SARS-CoV-2 (1 × 10^7^ pfu/mL) with 90 µl of vLAB (Pass 1, P1 sample). We incubated the sample for 10 or 20 min at room temperature and then we tested direct amplification using as template 2 µL of P1. In addition, we tested lysates from P2 (Table 1). As expected, we only had amplification in samples from P1 but not in negative samples (uninfected), which demonstrated the feasibility of direct amplification (Table 1). In P1 samples, we observed CT values of 31–33 in samples incubated for 10 min and over time the CT values increased by 2 cycles (36–35 for 20 min). These results suggest that samples stored at room temperature are susceptible to degradation over the time. This degradation could be explained due to residual activity of RNAses. Therefore, physical or chemical inactivation of RNAses must be conducted in clinical samples inactivated with vLAB.

**Table 1:** Direct amplification of SARS-CoV-2 in vLAB.

Gene	Ni	N2	Ni	N2	Ni	N2
	P1	P1	P1	P1	P1	P1
(A)	Neg	Neg	65 °C	65 °C	95 °C	95 °C
CT rep 1	Neg	Neg	19.39	18.59	19.07	18.86
CT rep 2	Neg	Neg	19.48	19.03	19.66	18.93
CT rep 3	Neg	Neg	19.22	19	19.54	18.55
CT Avg	Neg	Neg	19.36	18.87	19.42	18.78
± SD	Neg	Neg	0.13	0.24	0.31	0.20

	P2	P2	P2	P2	P2	P2
(B)	Neg	Neg	65 °C	65 °C	95 °C	95 °C
CT rep 1	Neg	Neg	Neg	Neg	Neg	Neg
CT rep 2	Neg	Neg	Neg	Neg	Neg	Neg
CT rep 3	Neg	Neg	Neg	Neg	Neg	Neg
CT Avg	Neg	Neg	Neg	Neg	Neg	Neg
± SD	Neg	Neg	Neg	Neg	Neg	Neg

(A) Pass1 (P1): Ct values from direct RT-PCR amplification using as template 2 µL of lysates of SARS-CoV-2 virus in vLAB (diluted 1:10). The samples were incubated at room temperature (RT) for 10 (light green) and 20 min (dark green) and then were tested by RT-PCR. Neg = non detected, only MEM culture media. (B) Pass 2 (P2): CT values from direct RT-PCR amplification using as template 2 µL of supernatants from P1, samples were incubated as described previously. Neg = Non detected, Rep = repetition, CT = cycle thresholds, N1 and N2 = nucleocapsid gene 1 and 2. vLAB = virus Lysis Amplification Buffer.

### Heated RNA of SARS-Cov-2 is amplified by dRT-PCR

**Figure 3:**
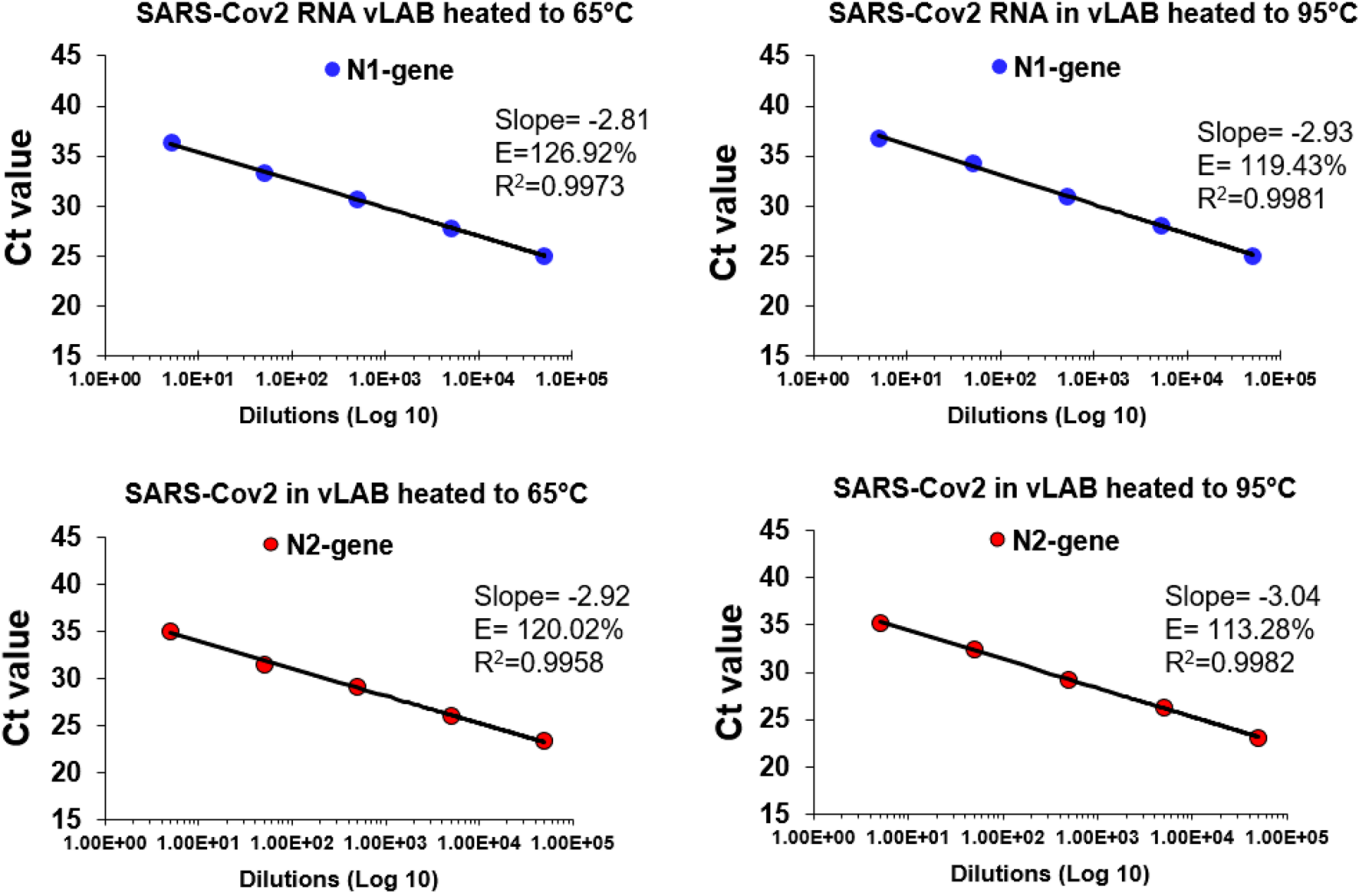
PCR efficiency of SARS-CoV-2 RNA in vLAB at 65 °C and 95 °C. PCR efficiency (E) for N1 and N2 gene was assessed using a duplicate tenfold dilution series of SARS-CoV-2 viral RNA.

We investigated the effect of temperature on dRT-PCR using samples of viral RNA diluted in vLAB as template. For these experiments, we incubated RNA spiked in vLAB at 65 °C for 10 min and 95 °C for 2 min. The results showed a slight variation in PCR efficiency in the samples incubated at room temperature (Fig. 3). However, overall, the limit of detection (5 copies per reaction) and CT values were not affected by high temperature and similar values to amplification at room temperature were observed. This result confirmed that vLAB and high temperature does not affect RNA integrity or downstream PCR amplification. Thus, it should be feasible to use heat for enzymatic inactivation on infected cells diluted in vLAB.

### SARS-CoV-2 is amplified in heat inactivated samples

We investigated the effect of temperature on dRT-PCR using infected cells in vLAB as template. We hypothesized that heat inactivation of RNAses reduces viral RNA degradation in samples inactivated with vLAB, therefore, incubation of samples in vLAB at high temperature should enhance PCR amplification. To test this hypothesis, we investigated the effect of high temperature on direct amplification using lysates of infected cells in vLAB as template. Samples heated at 65 °C × 10 min and 95 °C × 2 min showed similar CT values. However, these CT values are lower (Table 2) compared with values previously obtained in samples incubated at room temperature. Thus, amplification is more efficient in heated samples. This result confirms that incubation of samples obtained in vLAB at high temperatures enhance amplification of SARS-CoV-2 by RT-PCR. Therefore, enzymatic heat inactivation should be an included step to enhance sensitivity of the assay in clinical samples inactivated with vLAB.

**Table 2:** Direct amplification of SARS-CoV-2 in vLAB heated.

Gene	Ni	N2	Ni	N2	Ni	N2
	P1	P1	P1	P1	P1	P1
(A)	Neg	Neg	10 min	10 min	20 min	20 min
CT rep 1	Neg	Neg	33.03	31.7	35.22	35.78
CT rep 2	Neg	Neg	33.43	31.03	37.27	35.54
CT rep 3	Neg	Neg	33.5	32.06	38.37	36.26
CT Avg	Neg	Neg	33.32	31.59	36.95	35.86
± 50	Neg	Neg	0.25	0.52	1.59	0.36

	P2	P2	P2	P2	P2	P2
(B)	Neg	Neg	10 min	10 min	20 min	20 min
CT rep 1	Neg	Neg	Neg	Neg	Neg	Neg
CT rep 2	Neg	Neg	Neg	Neg	Neg	Neg
CT rep 3	Neg	Neg	Neg	Neg	Neg	Neg
CT Avg	Neg	Neg	Neg	Neg	Neg	Neg
± 50	Neg	Neg	Neg	Neg	Neg	Neg

(A) Pass1 (P1): Ct values from direct RT-PCR amplification using as template 2 µl of lysates of SARS-CoV-2 virus in vLAB (diluted 1:10) heated. The samples were incubated at 65 °C for 10 (light red) and 65 °C for 2 min (orange) and then were tested by RT-PCR. Neg = non detected, only MEM culture media. (B) Pass 2 (P2): CT values from direct RT-PCR amplification using as template 2 µl of supernatants from P1, samples were incubated as described previously. Neg = Non detected, Rep = repetition, CT = cycle thresholds, N1 and N2 = nucleocapsid gene 1 and 2. vLAB = virus Lysis Amplification Buffer.

### SARS-CoV-2 is detected in clinical samples diluted in vLAB

Since the goal of this preliminary work was to demonstrate the feasibility to conduct direct amplification of COVID-19 samples, we next tested direct amplification in heat-inactivated clinical samples that were diluted in vLAB. We used 30 nasopharyngeal clinical samples previously tested by RT-PCR at UTMB hospital (15 positive and 15 negative). All nasopharyngeal samples were obtained with standard protocols using swabs and placed in viral transport media. For direct RT-PCR, we inactivated samples using 90 µL of sample and 10 µL of a concentrated solution of 10 × vLAB. After dilution, samples were incubated 65 °C for 10 min and then placed on ice until use; Two microliters of the lysates were used for RT-PCR detection following the CDC protocol. To evaluate direct RNA amplification, we compared CT values from dRT-PCR vs CT values of clinical samples (previously tested in UTMB clinical laboratory), however for dRT-PCR we isolated RNA with the QIAmp DSP Viral RNA Mini Kit (Table 3). Our results showed 100% correlation for detecting positive and negative samples comparing QIAmp extraction vs vLAB, thus, we detected 15 positive and 15 negative samples with both methods. We observed a slight variation on CT values for both methods, however, this variation was mainly observed in samples with low CT values (samples with high-moderate viral load, *e.g.* sample #27, 16, and 24). Thus, this variation does not affect the qualitative results. In other experiments, we conducted a blinded study to evaluate the performance of dRT-PCR in clinical samples (previously tested at UTMB clinical laboratory) with high and low viral loads (Table 4). In these experiments, the dRT-PCR in vLAB method detected 100% of the positive and negative samples with high loads (< 30 CT) and only 1 positive sample with low viral load was not detected since the CT value was out of the limit of detection (CT 40). This result demonstrates the feasibility to use concentrated vLAB for direct amplification of SARS-CoV-2 in heat-inactivated samples. Importantly, we realize that validation of this method with a larger number of clinical samples is needed.

**Table 3:** Direct amplification of clinical samples in vLAB.

Sample	Lab result	CT N1	CT N2	CT N1	CT N2
1	Neg	ND	ND	ND	ND
2	Neg	ND	ND	ND	ND
3	Neg	ND	ND	ND	ND
4	Neg	ND	ND	ND	ND
5	Neg	ND	ND	ND	ND
6	Neg	ND	ND	ND	ND
7	Neg	ND	ND	ND	ND
8	Neg	ND	ND	ND	ND
9	Neg	ND	ND	ND	ND
10	Neg	ND	ND	ND	ND
11	Neg	ND	ND	ND	ND
12	Neg	ND	ND	ND	ND
13	Neg	ND	ND	ND	ND
14	Neg	ND	ND	ND	ND
15	Neg	ND	ND	ND	ND
16	Pos	27.7	27.5	31.5	31.2
17	Pos	34.1	33.03	36.0	35.5
18	Pos	29.8	28.5	29.4	29.5
19	Pos	37.2	38.0	37.4	36.9
20	Pos	26.2	25.9	25.3	24.7
21	Pos	32.1	32.5	38.9	37.2
22	Pos	34.9	33.4	38.7	37.8
23	Pos	29.5	29.6	30.0	30.2
24	Pos	29.2	29.0	31.1	32.2
25	Pos	37.1	37.0	37.8	36.9
26	Pos	38.2	37.9	38.8	37.8
27	Pos	19.2	19.1	17.9	17.7
28	Pos	38.0	36.0	38.2	37.5
29	Pos	37.9	37.7	36.9	36.3
30	Pos	36.0	35.09	35.8	35.0

Clinical samples from positive (Pos) and negative (Neg) patients were purified with QIAGEN RNA extraction kit or diluted with vLAB. The CT values from RT-PCR amplification with RNA extracted with QIAGEN and vLAB.

**Table 4:** RT-PCR vs dRT-PCR with vLAB.

RT-PCR			dRT-PCR			
Sample	CT	Method	Sample	CT NP1	CT NP2	Method
15	22.5	Fusion	15	15.7	15.1	vLAB
20	25	Fusion	20	22.4	22.5	vLAB
21	13.5	Fusion	21	17.6	16.5	vLAB
29	21.6	Fusion	29	34.6	32.1	vLAB
31	15.3	Fusion	31	21.9	30.2	vLAB
39	22	Fusion	39	19.84	20.2	vLAB
114	35.7	Fusion	114	36.4	34.8	vLAB
115	36.1	Fusion	115	37.9	36.5	vLAB
116	33	Fusion	116	36.13	34.2	vLAB
117	32.2	Fusion	117	32.6	31	vLAB
119	33.8	Fusion	119	35.66	35.2	vLAB
120	36.5	Fusion	120	34.6	36.2	vLAB
122	37	Fusion	122	40.4	42.1	vLAB
152	22.5	Fusion	152	34.4	30.5	vLAB
153	22.2	Fusion	153	27.9	25.7	vLAB
154	16.9	Fusion	154	21.67	21.02	vLAB
155	21.4	Fusion	155	29.34	27.6	vLAB
156	28.6	Fusion	156	34.14	31.4	vLAB
157	Negative	Fusion	157	Neg	Neg	vLAB
158	Negative	Fusion	158	Neg	Neg	vLAB
159	Negative	Fusion	159	Neg	Neg	vLAB
160	Negative	Fusion	160	Neg	Neg	vLAB
161	Negative	Fusion	161	Neg	Neg	vLAB
162	Negative	Fusion	162	Neg	Neg	vLAB
163	Negative	Fusion	163	Neg	Neg	vLAB
164	Negative	Fusion	164	Neg	Neg	vLAB
165	Negative	Fusion	165	Neg	Neg	vLAB
166	Negative	Fusion	166	Neg	Neg	vLAB
167	Negative	Fusion	167	Neg	Neg	vLAB
168	Negative	Fusion	168	Neg	Neg	vLAB
169	Negative	Fusion	169	Neg	Neg	vLAB
170	Negative	Fusion	170	Neg	Neg	vLAB
171	Negative	Fusion	171	Neg	Neg	vLAB

CT values of positive samples purified with fusion (left column) were compared with CT values of samples in vLAB amplified by dRT-PCR (right column).

## Discussion

We showed that a viral lysis amplification buffer (vLAB) prepared with IGEPAL allows dRT-PCR amplification of SARS-CoV-2 using primers and protocols approved by the CDC. The COVID-19 pandemic has resulted in an unprecedented worldwide demand for PCR testing. The huge increase in molecular testing has resulted in shortages of PCR reagents, viral transport media (VTM), and viral RNA extraction kits. This problem is exacerbated mainly in undeveloped countries and remote areas where the supply chain for reagents is inefficient. Our goal in this work was to test inexpensive alternatives for molecular detection of SARS-CoV-2 that could be implemented in these low resource settings.

We considered that dRT-PCR amplification could be a low-cost alternative for SARS-CoV-2 detection since this technique does not require RNA extraction kits nor specialized equipment for extraction. Currently, there are several commercial reagents for dRT-PCR, however, we decided to test an inexpensive protocol reported by Shatzkes et al.^10^. This method is based on the use of a lysis amplification buffer prepared with IGEPAL-630 (octylphenoxypolyethoxyethanol). This reagent is a nonionic, non-denaturing detergent that has been used for solubilization, isolation, and purification of membrane protein complexes^9,10^. Since IGEPAL is a mild detergent that induces cellular lysis but does not inhibit PCR enzymes^9,10^, we hypothesized that vLAB (prepared with IGEPAL) would be optimal for detecting SARS-CoV-2 RNA by dRT-PCR. Our initial studies showed that vLAB components do not inhibit activity of reverse transcriptase and DNA polymerase included in the tested amplification kit since we did not observe differences between RNA spiked in vLAB or water (Fig. 1). Our initial studies showed that a vLAB component does not affect activity of reverse transcriptase and DNA polymerase included in the amplification kit (Fig. 1). To date, CDC has approved more than 10 RT-PCR kits for COVID detection (https://www.fda.gov/media/134922/download). These kits should also be individually tested for dRT-PCR with vLAB. However, since enzymatic activities and amplification reagents included in all approved kits are similar, we anticipate that there will not be significant differences.

RNA preservation after sample collection is essential to maximize sensitivity and specificity of the detection assay. To prevent degradation, we evaluated two heating temperatures with the objective to inhibit RNAase activity. Both tested temperatures did not affect amplification of spiked RNA in the vLAB samples. Recent studies have demonstrated that virus inactivation is achieved at 70 °C and here we confirmed heat inactivation in infected cells which were lysed and diluted in vLAB. In addition, we demonstrated the feasibility of conducting dRT-PCR amplification in these samples. The vLAB inactivation will allow sample handling in BSL-2 laboratories, thereby reducing the exposure risk of personnel. Clinical samples used in this study were collected in VTM (~ 3 ml), to enhance viral concentration in the sample, and we used 9 parts of VTM and 1 part of a concentrated solution (10x) of vLAB. Our initial assessment with clinical samples showed a 100% correlation in dRT-PCR with vLAB and RT-PCR conducted with RNA purified in our laboratory with a commercial kit, however, we observed variation in CT values that may reflect differences in viral load or sample degradation. To address this question, we conducted additional experiments to evaluate performance of dRT-PCR in samples with differences in viral load. We found that in samples with high viral load (Low CT values < 30) we obtained 100% correlation, however, the sensitivity was slightly reduced in samples with high CTs. These results suggest that slight variations in CT values may be due to sample degradation during transportation or multiple thawing. A larger experiment with fresh samples should be conducted to validate dRT-PCR results for samples that are close to the limit of detection.

Overall, our results verified that using vLAB for molecular diagnostics of SARS-CoV-2 is a feasible method that should be pursued. Since IGEPAL-630 is an inexpensive reagent, the protocol described here could represent an affordable alternative for developing countries or remote areas for molecular detection of SARS-CoV-2.

## Supplementary Information﻿

Supplementary Figure 1.

## Data Availability

Raw data supporting the findings of this study are available from the corresponding author upon reasonable request.

## References

[1] Sharma A, Tiwari S, Deb MK, Marty JL (2020). Severe acute respiratory syndrome coronavirus-2 (SARS-CoV-2): A global pandemic and treatment strategies. Int. J. Antimicrob. Agents..

[2] Peck KR (2020). Early diagnosis and rapid isolation: Response to COVID-19 outbreak in Korea. Clin. Microbiol. Infect..

[3] Udugama B, Kadhiresan P, Kozlowski HN, Malekjahani A, Osborne M, Li VYC, Chen H, Mubareka S, Gubbay JB, Chan WCW (2020). Diagnosing COVID-19: The disease and tools for detection. ACS Nano.

[4] D'Cruz RJ, Currier AW, Sampson VB (2020). Laboratory testing methods for novel severe acute respiratory syndrome-coronavirus-2 (SARS-CoV-2). Front. Cell. Dev. Biol..

[5] Smithgall MC, Dowlatshahi M, Spitalnik SL, Hod EA, Rai AJ (2020). Types of assays for SARS-CoV-2 testing: A review. Lab. Med..

[6] Lu X, Wang L, Sakthivel SK, Whitaker B, Murray J, Kamili S, Lynch B, Malapati L, Burke SA, Harcourt J, Tamin A, Thornburg NJ, Villanueva JM, Lindstrom S (2020). US CDC real-time reverse transcription PCR panel for detection of severe acute respiratory syndrome coronavirus 2. Emerg. Infect. Dis..

[7] Uhteg K, Jarrett J, Richards M, Howard C, Morehead E, Geahr M, Gluck L, Hanlon A, Ellis B, Kaur H, Simner P, Carroll KC, Mostafa HH (2020). Comparing the analytical performance of three SARS-CoV-2 molecular diagnostic assays. J. Clin. Virol..

[8] Esbin MN, Whitney ON, Chong S, Maurer A, Darzacq X, Tjian R (2020). Overcoming the bottleneck to widespread testing: A rapid review of nucleic acid testing approaches for COVID-19 detection. RNA.

[9] Le AV, Huang D, Blick T, Thompson EW, Dobrovic A (2015). An optimised direct lysis method for gene expression studies on low cell numbers. Sci. Rep..

[10] Shatzkes K, Teferedegne B, Murata H (2014). A simple, inexpensive method for preparing cell lysates suitable for downstream reverse transcription quantitative PCR. Sci. Rep..

[11] Zhang Z, Kermekchiev MB, Barnes WM (2010). Direct DNA amplification from crude clinical samples using a PCR enhancer cocktail and novel mutants of Taq. J. Mol. Diagn..

[12] Kim Y, Lee WN, Yoo HJ, Baek C, Min J (2017). Direct buffer composition of blood pre-process for nucleic acid based diagnostics. Biochip. J..

[13] Svec D, Andersson D, Pekny M, Sjoback R, Kubista M, Stahlberg A (2013). Direct cell lysis for single-cell gene expression profiling. Front. Oncol..

[14] Batejat C, Grassin Q, Manuguerra JC, Leclercq I (2021). Heat inactivation of the severe acute respiratory syndrome coronavirus 2. J. Biosaf. Biosecur..

