# Supplementary material for: Direct RT-PCR amplification of SARS-CoV-2 from clinical samples using a concentrated viral lysis-amplification buffer prepared with IGEPAL-630
Source: Sci Rep. 2021 Jul 9;11:14204. doi: 10.1038/s41598-021-93333-2 (PMC8270935; doi:10.1038/s41598-021-93333-2)
Supplement: Supplementary file 1 — Supplementary Figure 1. [file 41598_2021_93333_MOESM1_ESM.docx]

Supplementary Data


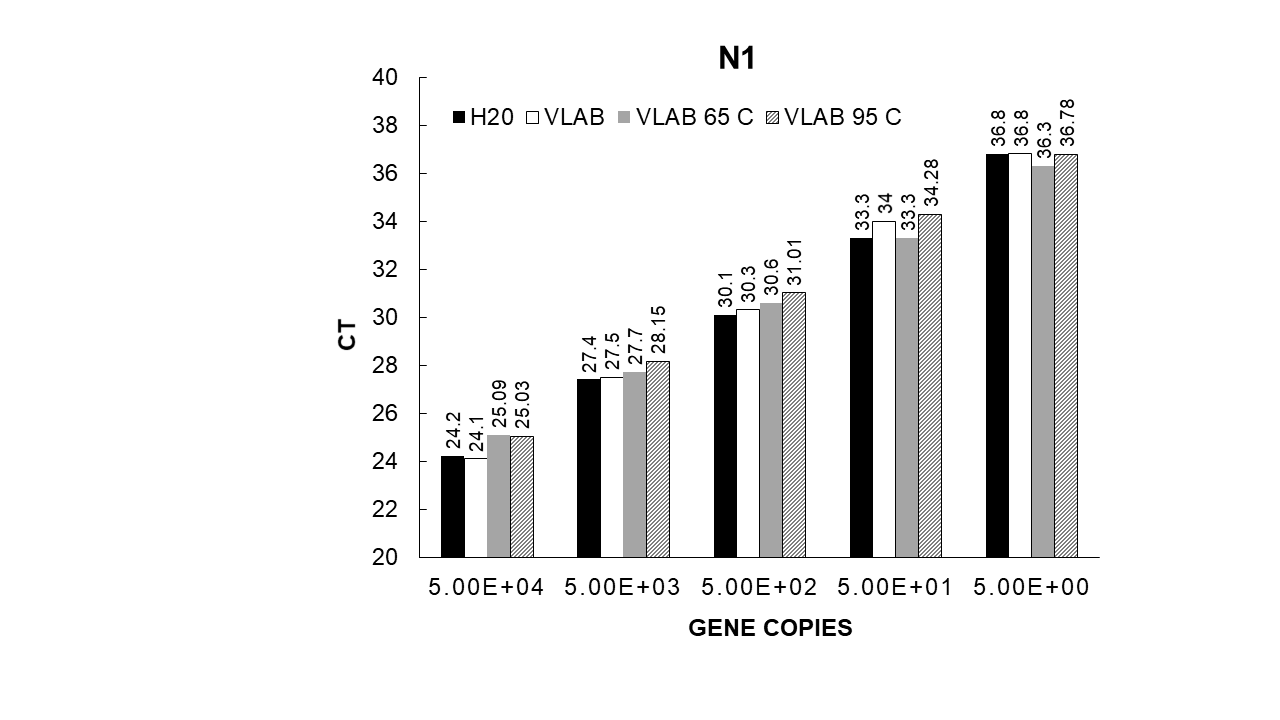
Supplemental figure 1.

**B**

**A**


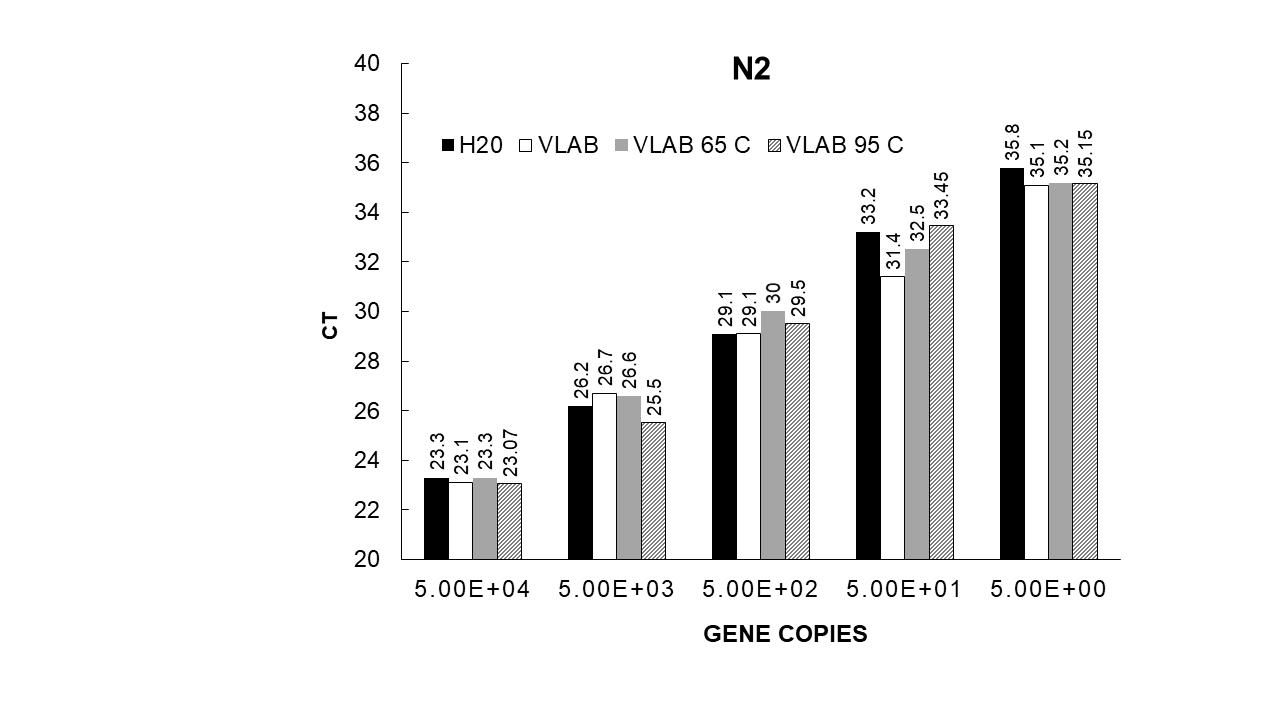


Supplemental figure S1. Amplification of SARS-Cov-2 RNA diluted in water and vLAB. Comparison of CT values from RT-PCR of N1 (A) and N2 (B) genes using as template RNA samples incubated at room temperature (black and white) of 65°C (grey) and 95°C (grey lined).
